# MOCOnet: Robust Motion Correction of Cardiovascular Magnetic Resonance T1 Mapping Using Convolutional Neural Networks

**DOI:** 10.3389/fcvm.2021.768245

**Published:** 2021-11-23

**Authors:** Ricardo A. Gonzales, Qiang Zhang, Bartłomiej W. Papież, Konrad Werys, Elena Lukaschuk, Iulia A. Popescu, Matthew K. Burrage, Mayooran Shanmuganathan, Vanessa M. Ferreira, Stefan K. Piechnik

**Affiliations:** ^1^Oxford Centre for Clinical Magnetic Resonance Research (OCMR), Division of Cardiovascular Medicine, Radcliffe Department of Medicine, University of Oxford, Oxford, United Kingdom; ^2^Nuffield Department of Population Health, University of Oxford, Oxford, United Kingdom; ^3^Big Data Institute, Li Ka Shing Centre for Health Information and Discovery, University of Oxford, Oxford, United Kingdom

**Keywords:** cardiovascular magnetic resonance, deep learning, image registration, ShMOLLI, T1 mapping

## Abstract

**Background:** Quantitative cardiovascular magnetic resonance (CMR) T1 mapping has shown promise for advanced tissue characterisation in routine clinical practise. However, T1 mapping is prone to motion artefacts, which affects its robustness and clinical interpretation. Current methods for motion correction on T1 mapping are model-driven with no guarantee on generalisability, limiting its widespread use. In contrast, emerging data-driven deep learning approaches have shown good performance in general image registration tasks. We propose MOCOnet, a convolutional neural network solution, for generalisable motion artefact correction in T1 maps.

**Methods:** The network architecture employs U-Net for producing distance vector fields and utilises warping layers to apply deformation to the feature maps in a coarse-to-fine manner. Using the UK Biobank imaging dataset scanned at 1.5T, MOCOnet was trained on 1,536 mid-ventricular T1 maps (acquired using the ShMOLLI method) with motion artefacts, generated by a customised deformation procedure, and tested on a different set of 200 samples with a diverse range of motion. MOCOnet was compared to a well-validated baseline multi-modal image registration method. Motion reduction was visually assessed by 3 human experts, with motion scores ranging from 0% (strictly no motion) to 100% (very severe motion).

**Results:** MOCOnet achieved fast image registration (<1 second per T1 map) and successfully suppressed a wide range of motion artefacts. MOCOnet significantly reduced motion scores from 37.1±21.5 to 13.3±10.5 (*p* < 0.001), whereas the baseline method reduced it to 15.8±15.6 (*p* < 0.001). MOCOnet was significantly better than the baseline method in suppressing motion artefacts and more consistently (*p* = 0.007).

**Conclusion:** MOCOnet demonstrated significantly better motion correction performance compared to a traditional image registration approach. Salvaging data affected by motion with robustness and in a time-efficient manner may enable better image quality and reliable images for immediate clinical interpretation.

## 1. Introduction

Quantitative T1 mapping is a novel approach in cardiovascular magnetic resonance (CMR) for myocardial tissue characterisation (1). Native and post-contrast T1 mapping offer quantitative, pixel-wise measures to detect tissue changes in myocardial composition (2) and have been used in the assessment of myocardial inflammation (3), oedema (4, 5), infiltration (6), diffuse fibrosis (7), and other pathologies (8). Stress T1 mapping has the potential to assess coronary artery disease without the need for gadolinium-based contrast agents (9–11).

T1 mapping is obtained from pixel-wise exponential recovery curve fitting of multiple T1-weighted images. With advances made from the original Look-Locker spectroscopic method (12), current mapping techniques employ intermittent image acquisition using electrocardiographic gating during multiple heartbeats (2). The shortened modified Look-Locker inversion recovery (ShMOLLI) (13) allowed shorter breath-holds with 9 heartbeats with high precision and reproducibility. Although acquiring multiple T1-weighted images at the same cardiac phase largely reduces the influence of cardiac motion, undesired respiratory motion still poses significant challenges (14). Uncorrected and unrecognised respiratory motion artefacts may cause errors in T1 estimation and incorrect diagnoses (13).

Retrospective motion correction (MOCO) on the multiple T1-weighted images can significantly improve the robustness and clinical utility of mapping techniques (15). Such correction is accomplished by aligning the T1-weighted images before reconstruction. The main challenge is the variation in image contrast and signal nulling of the multiple T1-weighted images acquired at different inversion times. Model-driven registration methods for MOCO were developed to circumvent this limitation with promising results (16–19). However, careful inspection for uncorrected residual motion or distortions from failures in motion correction is still needed (20). Although visual assessment in CMR is still the clinical standard for image interpretation (21), constant and long manual labour is prone to error due to inconsistency and operator fatigue, as well as slow clinical workflow if handling a large volume of images.

With the advent of deep learning, convolutional neural networks (CNN) have enabled unprecedented progress in image processing, shifting the paradigm from predefined, hand-crafted rules to automated learning procedures aided by large data. The rapid adaptation of deep learning approaches within CMR provides fast, consistent, and accurate pipelines primarily for image segmentation and analysis (22) significantly reducing physician labour hours. The field of clinical image registration with deep learning is also primed to replace iterative registration methods, with potential to improve accuracy, time efficiency and quality control (23), and applicability to cover the unmet need of MOCO in T1 maps. We hypothesised that a data-driven method for myocardial motion correction would suppress motion artefacts with more robustness and generalisability to serve large clinical datasets.

In this work, we present MOCOnet, a novel deep learning approach for myocardial motion correction developed using CMR T1 mapping from the UK Biobank (24). We adapted an encoder-decoder architecture with warping layers to aid the learning of such deformation in a coarse-to-fine manner. Given a set of T1-weighted images, MOCOnet can predict the deformation required to correct any present motion artefacts in a time-efficient manner. MOCOnet was tested for its motion correction performance against a well-validated multi-modal image registration method, using multiple blinded expert observers to validate the motion correction effectiveness.

## 2. Materials and Methods

### 2.1. Cardiac T1 Mapping and Motion Artefact

**Figure 1:**
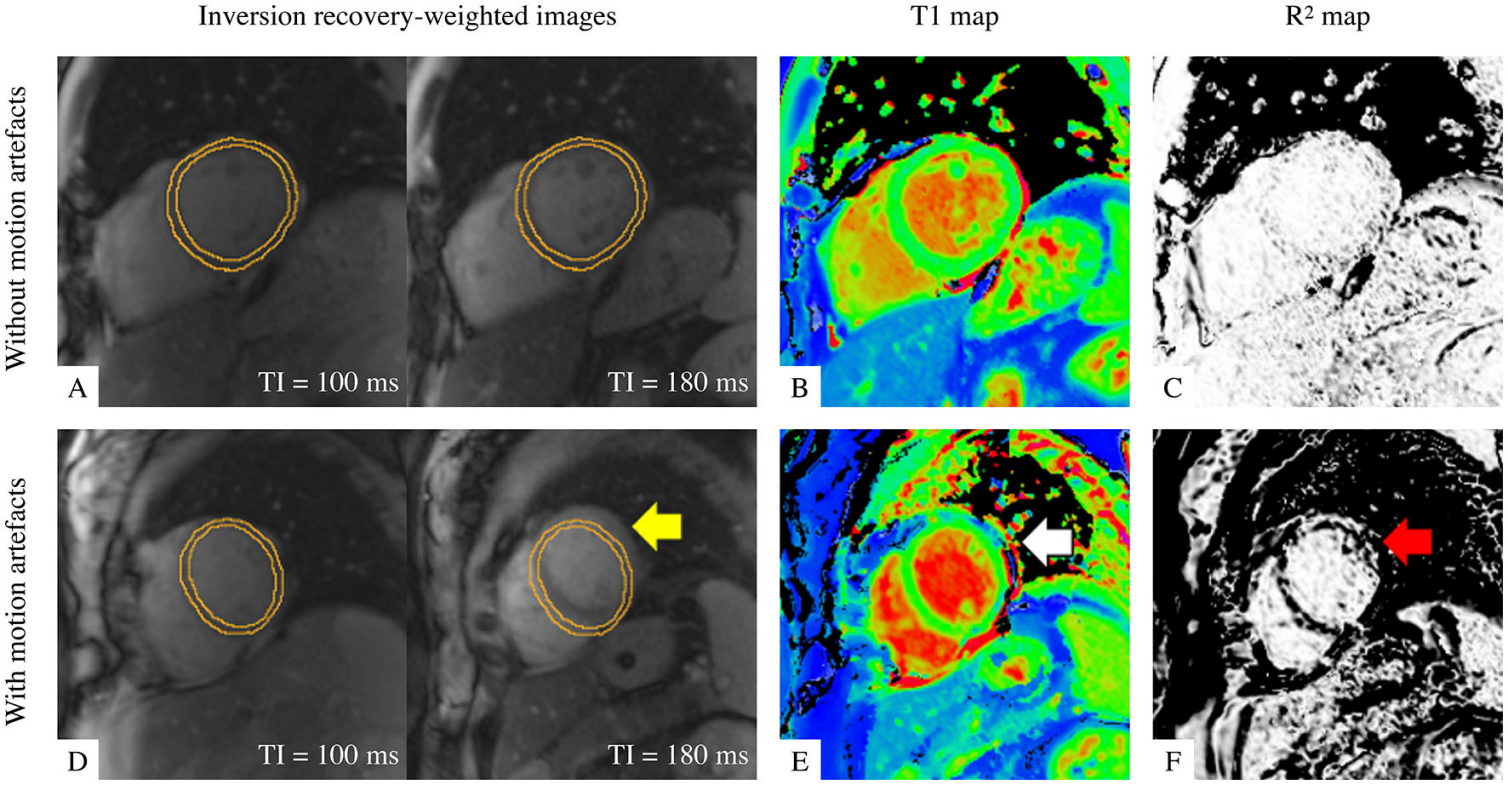
Illustration of T1 maps with good quality (top row) and with motion artefact (bottom row). **(A,D)** Two examples out of seven of inversion-recovery weighted (IRW) images required for T1 map reconstruction are shown, time-stamped with their corresponding inversion times (TI) and overlaid by identical manual myocardial contours for identifying motion. **(B,E)** ShMOLLI T1 maps. **(C,F)** R^2^ quality control maps. A good quality T1 map is indicated by **(A)** myocardium in same position and **(C)** “all white” in the left ventricular myocardium indicating high T1 fitting confidence. A T1 map with motion artefact is evident by misalignment in IRW images (yellow arrow), suspicious features in T1 map (white arrow) and dark bands in R^2^ map in the left ventricular myocardium as evidence of poor T1 fitting (red arrow).

Cardiac ShMOLLI T1 mapping is calculated by fitting exponential recovery curves to 7 inversion recovery-weighted (IRW) images with multiple inversion times (Figure 1A) and acquired within a short 9-heartbeat single breath-hold (13). The reconstructed T1 map (Figure 1B) enables pixel-wise quantification of T1 values. The associated map of coefficient of explained variance (R^2^ map; Figure 1C) allows quality monitoring of the curve fitting in reference to a mono-exponential T1 relaxation recovery model. A closer proximity to the reference displays a uniform white appearance of relevant regions of interest in the R^2^ map, whereas motion in the IRW images (Figure 1D, arrowed) decreases the T1 map interpretability (Figure 1E, arrowed), corresponding to the dark bands at the motion-affected areas in the R^2^ map (Figure 1F, arrowed). Besides motion artefacts, the R^2^ map is also sensitive to off-resonance, fat inclusion, mistriggering, and other artefacts (5, 25).

### 2.2. Non-rigid Registration Approach

Given that a T1 map with motion artefacts is composed of 7 unaligned IRW images, a motion-corrected T1 map can be achieved by aligning the IRW images. The motion artefact can be synthesised as a deformation of aligned IRW images with a displacement vector field (DVF). The non-rigid registration problem is then solved by estimating the inverse DVF of a given set of unaligned IRW images.

### 2.3. Multi-Scale Registration Neural Network

**Figure 2:**
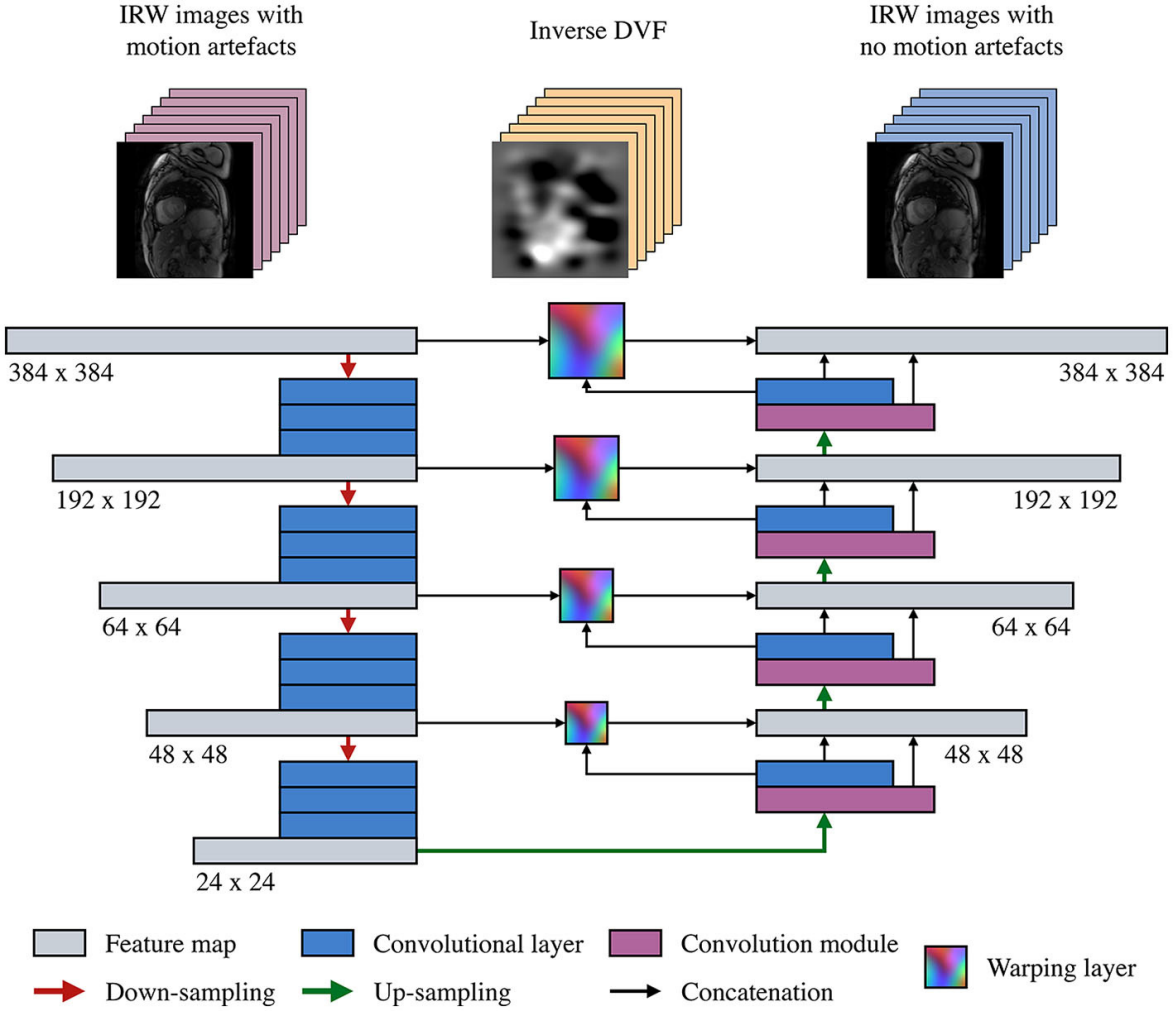
Structure of the proposed motion correction convolutional neural network (MOCOnet). A stack of seven inversion recovery-weighted (IRW) images is input into the encoder-decoder structure on a per-channel basis. The warping layers estimate the optical flow from all the channels in a coarse-to-fine manner at each scale. The last warping layer generates the inverse distance vector field (DVF), i.e., the deformation required to correct the motion artefacts, in a groupwise manner.

The proposed learning-based model corrects a T1 map by estimating the inverse DVF in each of its 7 IRW images to enable a non-rigid registration between them, before the T1 map reconstruction. The multi-scale registration CNN (Figure 2) adopts an encoder-decoder U-Net-like structure (26) and employs warping layers (27) between the contracting and expansive paths at each scale. The feature maps are down-sampled with a series of 3 × 3 convolutional layers followed by a batch normalisation layer, a leaky rectified linear unit and a max-pooling layer, and similarly up-sampled with a transposed convolutional layer. The warping layers speed up the training by imposing a loss function on a multi-scale manner and increase the registration accuracy by correcting motion starting from coarse levels and passing the residual motion to higher resolution layers for fine motion correction.

The IRW images are first fed in a sequence of convolution and downsampling operations to produce features at multiple scales on a per-channel basis. The features, from low to high resolutions, are then used as input of convolution modules to produce DVFs. Each convolution module takes as input the features from the previous step, the DVF at the previous scale, and the warped features from the downsampling stage. Applying warping at each of the 4 scales enables the use of residual motion information to be corrected and refined in the next scale. Hence, the neural network generates the DVFs in a coarse-to-fine manner and adds more details with higher resolution in each subsequent level, with a loss function defined at each scale to further supervise the learning manner.

### 2.4. Imaging Data and Inclusion Criteria

The imaging data comprised of over 5,000 CMR native T1 maps from the UK Biobank Imaging Component (24), acquired at the mid-ventricular short-axis view using the ShMOLLI T1 mapping sequence (13). For quality control, a trained human operator (EL), with over 10 years of experience in CMR image analysis, assessed the presence of any artefact in the left ventricular myocardium in the 7 IRW images for each T1 map. A total of 1,536 T1 maps were scored strictly as good quality with no artefact. The remaining data were marked to have either mild to severe motion or other imaging artefacts and were excluded from the training dataset. This strict quality control ensured that the neural network learnt to align the images accurately with no distraction from residual motion artefacts in the training data, i.e., with images that did not require any motion correction.

### 2.5. Training Procedure

**Figure 3:**
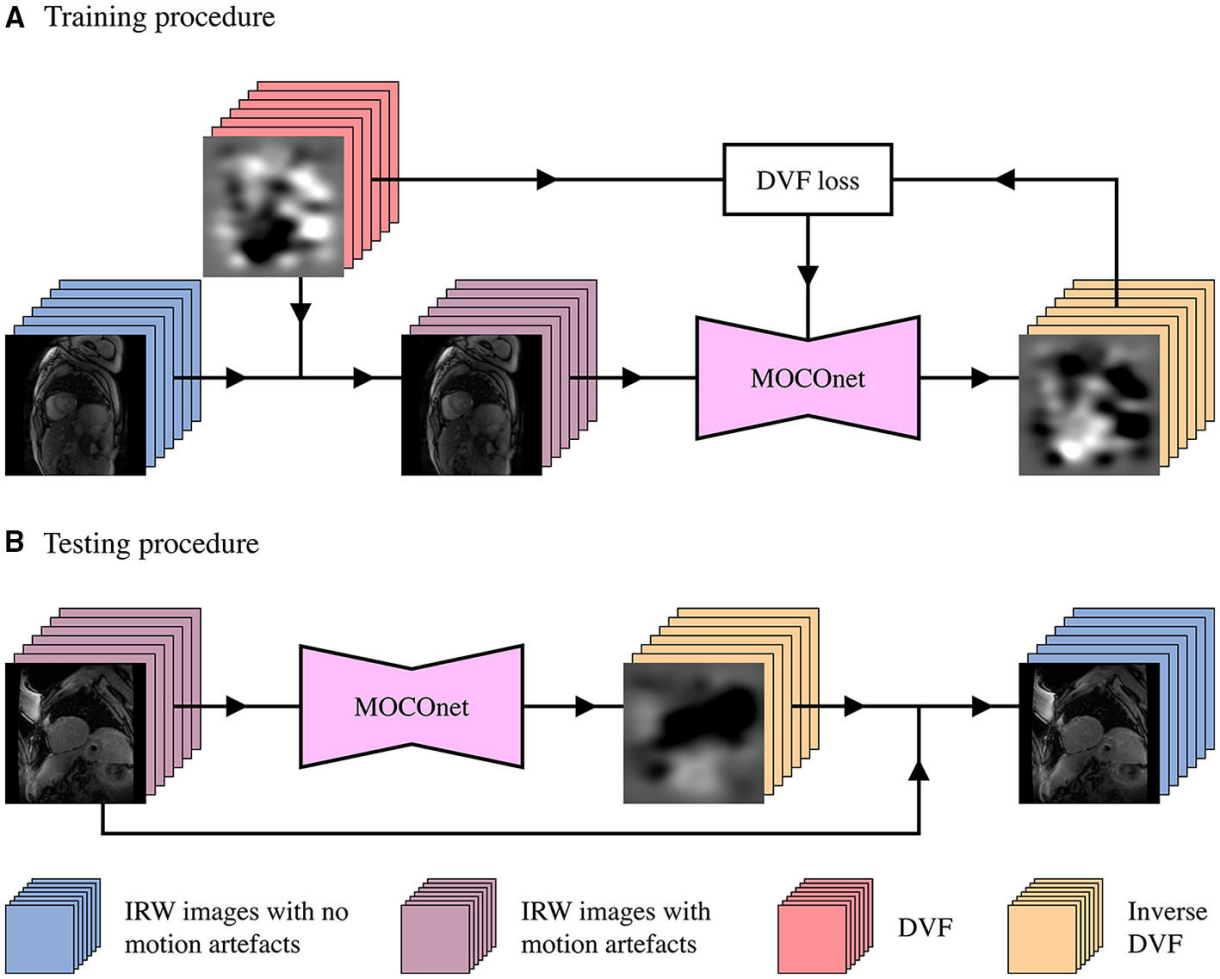
Development workflow of the proposed motion correction convolutional neural network (MOCOnet) for myocardial ShMOLLI T1 mapping. **(A)** MOCOnet was trained on 1,536 sets of seven inversion recovery-weighted (IRW) images with no motion artefacts which were synthetically deformed with displacement vector fields (DVFs), to predict the inverse DVF required to correct the motion. **(B)** MOCOnet was tested on 200 T1 maps with a varied degree of motion artefacts. Each stack denotes a set of seven images; each junction denotes the DVFs application to the IRW images; the box with DVF loss represents the weight adjustment during training.

The quality-controlled imaging data were used to generate a training dataset with 10% of the data preserved for validation. Artificial DVFs were generated as previously described (28) and applied to the IRW images without motion artefacts to synthesise random non-rigid motion without requiring segmentations (28). Specifically, 7 DVFs were generated with random parameters preserving anatomical topology. Mean displacement value at each pixel was calculated and removed from all 7 DVFs to focus on relative displacement between images. The generated DVFs were applied to each of the IRW images, respectively to produce deformed IRW images. The proposed model was trained to predict 7 inverse DVFs from 7 deformed IRW images with the synthetic, inverse DVFs as ground truth (Figure 3A).

### 2.6. Testing Procedure

Once trained, MOCOnet reads a given set of 7 IRW images with or without motion artefacts and estimates the deformation required to correct any present motion (Figure 3B), without ground truth. The T1 map is then reconstructed offline using motion-corrected images with an open source library for CMR parametric mapping (29).

### 2.7. Implementation Specification

All images were zero-padded to the same size of 384 × 384 pixels and image intensities were pre-processed with quantile normalisation to ensure generalisability (30). The multi-scale loss was calculated as the average mean square errors of the predicted DVFs at each scale and resolution. The neural network was optimised using the Adam method (31) with an initial learning rate of 0.001 and a learning rate scheduler to reduce the learning rate during the training, and mini-batch size of 4. Training was stopped once the validation loss did not decrease for 50 epochs. The network was trained for approximately 48 h until the training curve converged with low bias and variance using a NVIDIA TITAN XP GPU and implemented in TensorFlow (32). After the training, correcting motion for each set of 7 IRW images took less than 1 s on GPU or a modern CPU.

### 2.8. Validation

#### 2.8.1. Baseline Deformable Image Registration Method

The proposed method's performance was compared against a well-validated multi-modal image registration algorithm (33) as the baseline method. The registration method alleviated the problem of artificial motion discontinuities by combining a bilateral filter with an additional deformation field-based filter and a diffusion regularisation algorithm, serving as an excellent registration approach without requiring a prior image segmentation task as conventional methods. The baseline method, implemented in C, used the first IRW image as a reference image for all subsequent pairwise registrations and took approximately 30 s per T1 map on a modern CPU.

#### 2.8.2. Test on Respiratory Motion With Human Observer Scores

A multi-observer experiment was designed to evaluate the effectiveness and robustness of motion correction, and potential noise introduced to cases originally with no motion. From the UK Biobank, a test set of 200 real acquired T1 maps with various degree of motion artefacts was selected based on the existing quality scores by an experienced human observer. Specifically, 50 samples presented severe motion artefacts affecting all myocardial segments, 100 presented moderate motion affecting individual segments, and 50 presented mild to no motion.

The extent of motion on the test set was assessed in a 5-point categorical scale: ‘no motion’, ‘mild motion’, ‘moderate motion’, ‘severe motion’, and ‘very severe motion’, with a numerical scale between 0 to 100% behind the interface, to ensure both intuitiveness for human operators and practicality for statistical analyses. The baseline and proposed methods were applied to all samples unselectively, giving in total 400 motion-corrected samples. One hundred and twenty only samples (20%) were randomly chosen from the mixed 600 samples and duplicated to evaluate intra-observer variability. Three trained human observers (IP, MB and MS) were instructed to score the resultant 720 samples for the extent of motion. All observers were blinded to the original artefact scores and which motion correction method was applied. To reduce the variance of the human scores *X*_*i*_, the weighted average score $\bar{X}$ of the three observers (*i* = 3) was calculated as $\bar{X}=\sum W_{i}X_{i}/\sum W_{i}$. The weights *W*_*i*_ were calculated by the inverse of intra-observer variance σ_*i*_ (34, 35) based on the duplicated 20% cases, i.e., $W_{i}=1/\sigma_{i}^{2}$ for the *i*-th observer. The expected standard error of the weighted average scores was $SE\left(\bar{X}\right)={\sqrt{\sum W_{i}}}^{-1}$.

#### 2.8.3. Statistical Analysis

Quality scores were reported as mean ± standard deviation. Non-parametric Wilcoxon signed-rank test was used to assess the statistical difference between the data with and without motion correction by the baseline and proposed methods. Given the modest number of repeated comparisons within each group the statistical significance threshold was kept at standard *p* < 0.05 (36). Statistical analysis was performed using the Python programming language.

## 3. Results

**Figure 4:**
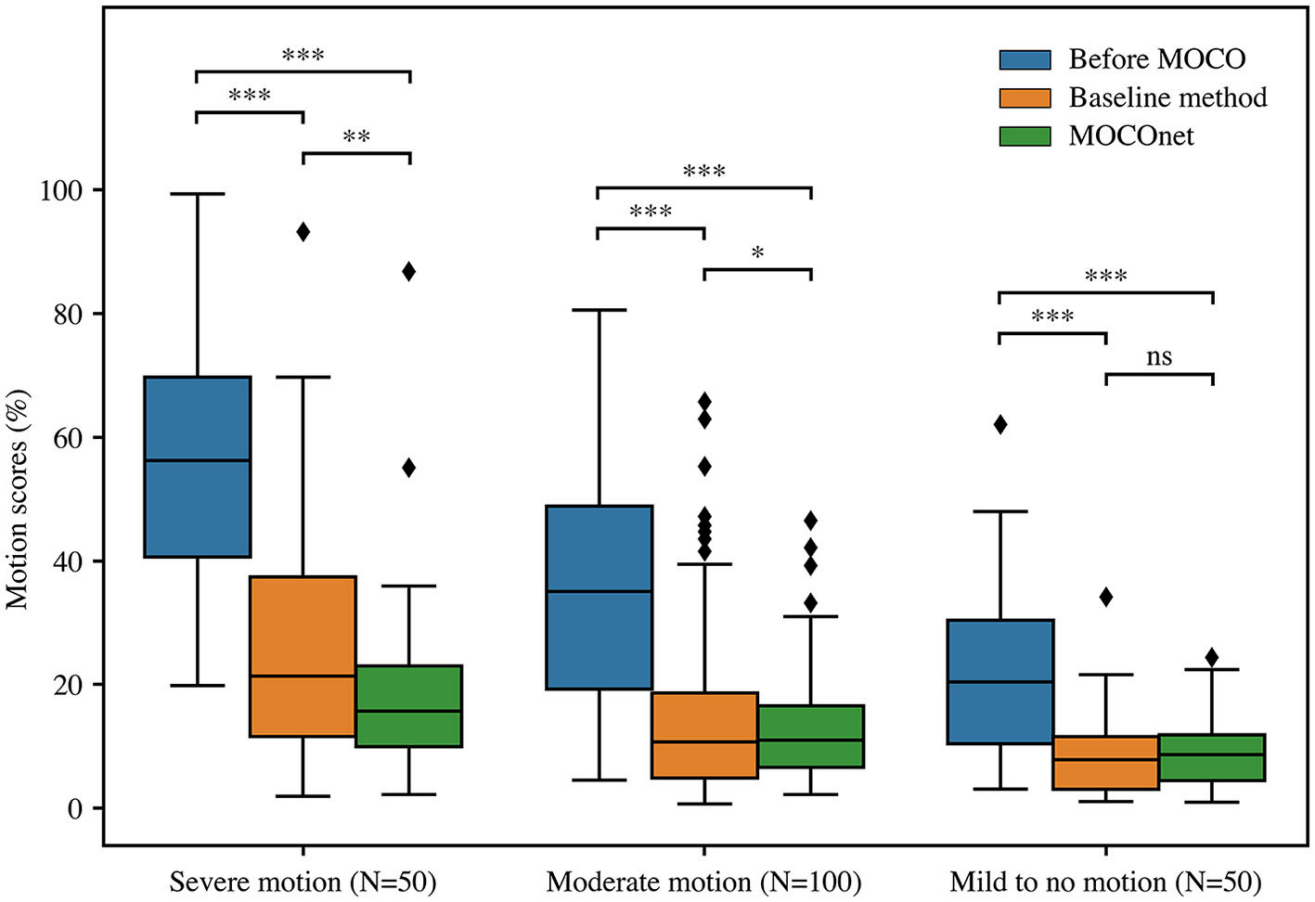
Motion correction (MOCO) performance of the baseline and the proposed deep learning-based motion correction (MOCOnet) methods. Box and whisker plot of motion scores in non-parametric terms of three data groups, before (blue) and after motion correction by the baseline (orange) and proposed MOCOnet (green) methods. Reported values are inverse variance-weighted scores of three human observers. MOCOnet achieved the best results and significantly reduced the motion artefacts. ^*^p = 0.04; ^**^p < 0.01; ^***^p < 0.001; ns = not significant.

The results of human observer validation on the 200 cases from the UK Biobank are reported in Table 1. Intra-observer variabilities of the three observers on the 20% duplicated cases were 10.6, 17.3 and 21.9, respectively. Standard error of the final weighted-average scores that were used to compare the motion correction methods was 8.3 at a scale from 0 to 100%. Overall, both methods significantly reduced the motion artefacts, from an average motion score of 37.1 ± 21.5 to 15.8 ± 15.6 (baseline method) and 13.3 ± 10.5 (MOCOnet; both *p* < 0.001). MOCOnet was significantly more effective at reducing motion artefacts than the baseline method for the subgroups with severe motion (*N* = 50, *p* = 0.006) and moderate motion (*N* = 100, *p* = 0.04). For the subgroup with mild to no motion (*N* = 50), both methods significantly further reduced the motion artefacts (both *p* < 0.001), and neither added noise, nor was significantly different from each other (*p* = 0.2). Overall, MOCOnet suppressed motion artefacts to a higher extent and in a more consistent way compared to the baseline method, as evidenced by its lower maximum score and variability (*N* = 200, *p* = 0.007). The boxplot of motion scores (Figure 4) further illustrates the above dependencies in non-parametric terms. This demonstrates that MOCOnet achieved a tighter span of perceived motion estimates, with better perceived robustness to outliers.

**Table 1:** Human observer assessment of motion extent (%) on 200 T1 maps before motion correction, and after the baseline and proposed method (MOCOnet) for motion correction.

	**All data** **(***N*** = 200)**	**Group 1** **(***N*** = 50) Severe motion**	**Group 2** **(***N*** = 100) Moderate motion**	**Group 3** **(***N*** = 50) Mild to no motion**
Before MOCO	37.1 ± 21.5	55.8 ± 18.7 (99.3)	35.5 ± 18.9 (80.5)	21.7 ± 13.8 (62.1)
Baseline method	15.8 ± 15.6	25.8 ± 19.8 (93.4)	14.7 ± 13.9 (65.7)	**8.1** ± 6.5 (34.2)
MOCOnet	**13.3 ± 10.5**	**18.6 ± 14.3 (86.9)**	**12.7 ± 9.2 (46.4)**	9.4 ± **6.4 (19.8)**

*The quality scores are inverse variance-weighted scores of three human observers and reported in mean ± SD (maximum value). The best results are highlighted in bold*.

**Figure 5:**
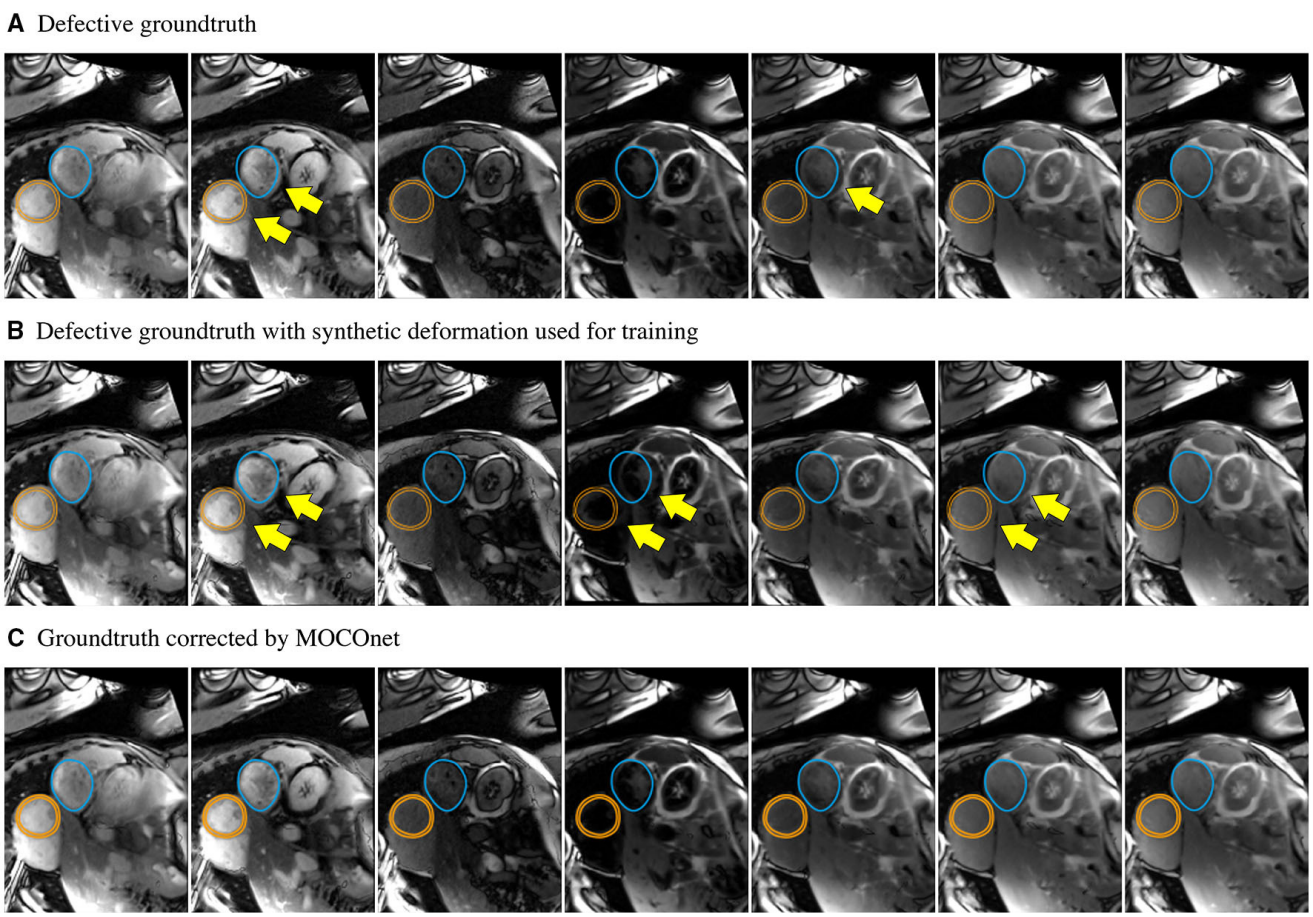
Robustness of the proposed motion correction convolutional neural network (MOCOnet) for myocardial ShMOLLI T1 mapping from a noisy training sample. **(A)** Training sample falsely considered free of motion (1 in 1,536) as manually depicted with unaligned myocardium (orange) and stomach (blue) with yellow arrows throughout the inversion recovery-weighted images. **(B)** Applied deformation to the training sample used for training. **(C)** Sample corrected by MOCOnet after training demonstrating the successful learning of the general rule without replicating the data.

MOCOnet successfully learnt from synthetic random motion to predict the required DVFs to correct the motion of IRW images ensuring a motion-corrected T1 map in real acquired data. Figure 5 exemplifies the robustness of the method. One training sample was falsely considered to have no motion artefacts, as evidenced by the overlaid contours of both myocardium and stomach but this did not overfit the learning or affect the final results. The data-driven process aided the learning of the general rule, as MOCOnet managed to correct the error in this training sample, instead of replicating it.

## 4. Discussion

In this work, MOCOnet, a novel end-to-end motion correction neural network for CMR T1 maps, was developed using a large-scale dataset and validated with expert human analysts. MOCOnet was able to automatically predict the deformation required to correct real motion artefact cases. The proposed method has a fast-processing speed of <1 s per T1 map and does not require modification of image acquisition sequences, external hardware, or user intervention, enabling direct implementation to clinical practise.

Although the principle of estimating the required DVFs on a given set of images to correct their mutual alignment was tested on myocardial ShMOLLI T1 maps, the problem formulation and solution are not limited to this mapping method or region of interest. The deformation estimation is alleviated by considering the images ‘as is’ with a data-driven procedure (37) without heavily relying on the differences in contrast, the specific inversion recovery times or a prior user input. This principle can be directly applied to other T1 mapping methods that require multiple T1-weighted images to be aligned in a groupwise manner to ensure an accurate exponential recovery curve fitting (38), to other organs that are evaluated with parametric mapping, such as the brain (39) and liver (40), and to other imaging modalities with varied image contrast (41).

The potential clinical impact of the method is promising. A large portion of the UK Biobank T1 mapping data analysed in this study presented mild to severe motion, hampering the diagnostic utility of T1 mapping. Although recent progress on automated motion artefact detection methods (42) may alleviate the quality monitoring process, rescanning to ensure a free-of-motion T1 map would increase scan times and reduce patient throughput. The presented data-driven MOCOnet approach provides an attractive solution to retrospectively suppress the motion using most of the acquired data to enhance T1 map quality, which is expected to salvage data corrupted by motion, reduce the need for rescanning and improve diagnosis. MOCOnet also holds promise for stress T1 mapping applications (9–11, 38) which may be subject to greater motion artefact. With the rapidly evolving field of deep learning, further research can be done to assess potential benefits of incorporating a more diverse variety of learning-based registration methods (23, 43) into a quality-control driven pipeline (44–46) to verify the registration accuracy on-the-fly including the R^2^ maps. With further work, MOCOnet together with T1 protocol quality assurance (47, 48) and automated myocardial segmentation (45) could ultimately lead to a comprehensive framework for robust T1 mapping for clinical use.

Despite a good performance in motion correction, as evidenced with the large improvement in the motion score, it is revealed by human observer experiments that MOCOnet could still fail in correcting images with severe motion. The challenge is not only due to difficulty in motion correction, but also through-plane motion, resulting in fitting of T1 values using signals at different tissue location. Breath holding remains crucial in acquiring good quality T1 maps. Future work will include validation on a multi-vendor, multi-centre population, expansion to other regions of interest, and direct implementation onto the scanner for robust inline motion artefact correction to generate good quality and reliable images for immediate clinical interpretation.

## 5. Conclusion

MOCOnet is an effective and robust convolutional neural network for correction of artefacts from myocardial motion. The technique can be readily deployed for post-processing of T1 mapping to restore T1 values in images affected by motion artefacts. This non-rigid registration solution can be further extended to other mapping methods, for generating good quality and reliable images for immediate clinical interpretation. MOCOnet can eventually enhance parametric mapping methods paving the way towards more reliable quantitative CMR medical imaging.

## Data Availability Statement

The imaging data were provided by the UK Biobank under the technical access agreement. Data access must be obtained directly from the UK Biobank.

## Author Contributions

RG, QZ, BP, and KW contributed to the design of the study. RG drafted the manuscript. QZ curated the database and analysed the results. EL quality controlled the UK Biobank data. IP, MB, and MS scored the motion extent. VF and SP provided research guidance and conceived the study. All authors critically edited and revised the article.

## Funding

RG acknowledges support for his D.Phil. studies from the Clarendon Fund, the Balliol College and the Radcliffe Department of Medicine, University of Oxford. QZ, VF, and SP acknowledge John Fell Oxford University Press Research Fund. QZ, MB, VF, and SP acknowledge support from the Oxford BHF Centre of Research Excellence (RE/18/3/34214). BP acknowledges Rutherford Fund at Health Data Research UK (MR/S004092/1). MB is supported by a British Heart Foundation (BHF) Clinical Research Training Fellowship (FS/19/65/34692). MS is supported by the Alison Brading Memorial Graduate Scholarship in Medical Science, Lady Margaret Hall, University of Oxford. IP, MB, VF, and SP acknowledge support from the National Institute for Health Research (NIHR) Oxford Biomedical Research Centre at The Oxford University Hospitals NHS Foundation Trust.

## Conflict of Interest

SP has patent authorship rights for U.S. patent 9285446 B2 systems and methods for Shortened Look-Locker Inversion Recovery ShMOLLI cardiac gated mapping of T1, granted March 15, 2016; licensed to Siemens Medical. KW is an employee of Circle Cardiovascular Imaging since 2019. The remaining authors declare that the research was conducted in the absence of any commercial or financial relationships that could be construed as a potential conflict of interest.

## Publisher's Note

All claims expressed in this article are solely those of the authors and do not necessarily represent those of their affiliated organizations, or those of the publisher, the editors and the reviewers. Any product that may be evaluated in this article, or claim that may be made by its manufacturer, is not guaranteed or endorsed by the publisher.

## References

[1] Karamitsos TD Arvanitaki A Karvounis H Neubauer S Ferreira VM. Myocardial tissue characterization and fibrosis by imaging. JACC Cardiovasc Imaging. (2020) 13:1221–34. 10.1016/j.jcmg.2019.06.030 31542534

[2] Messroghli DR Moon JC Ferreira VM Grosse-Wortmann L He T Kellman P. Clinical recommendations for Cardiovascular Magnetic Resonance mapping of T1, T2, T2^*^ and extracellular volume: A consensus statement by the Society for Cardiovascular Magnetic Resonance (SCMR) endorsed by the European Association for Cardiovascular Imaging (EACVI). J Cardiovasc Magn Reson. (2017) 19:75. 10.1186/s12968-017-0389-8 28992817 PMC5633041

[3] Ferreira VM Schulz-Menger J Holmvang G Kramer CM Carbone I Sechtem U. Cardiovascular magnetic resonance in nonischemic myocardial inflammation. J Am Coll Cardiol. (2018) 72:3158–3176. 10.1016/j.jacc.2018.09.072 30545455

[4] Messroghli DR Niendorf T Schulz-Menger J Dietz R Friedrich MG. T1 mapping in patients with acute myocardial infarction. J Cardiovasc Magn Reson. (2003) 5:353–9. 10.1081/JCMR-120019418 12765114

[5] Ferreira VM Piechnik SK Dall'Armellina E Karamitsos TD Francis JM Choudhury RP. Non-contrast T1-mapping detects acute myocardial edema with high diagnostic accuracy: a comparison to T2-weighted cardiovascular magnetic resonance. J Cardiovasc Magn Reson. (2012) 14:42. 10.1186/1532-429X-14-42 22720998 PMC3424120

[6] Karamitsos TD Piechnik SK Banypersad SM Fontana M Ntusi NB Ferreira VM. Noncontrast T1 Mapping for the diagnosis of cardiac amyloidosis. JACC Cardiovasc Imaging. (2013) 6:488–97. 10.1016/j.jcmg.2012.11.013 23498672

[7] Everett RJ Stirrat CG Semple SIR Newby DE Dweck MR Mirsadraee S. Assessment of myocardial fibrosis with T1 mapping MRI. Clin Radiol. (2016) 71:768–78. 10.1016/j.crad.2016.02.013 27005015

[8] Liu JM Liu A Leal J McMillan F Francis J Greiser A. Measurement of myocardial native T1 in cardiovascular diseases and norm in 1291 subjects. J Cardiovasc Magn Reson. (2017) 19:74. 10.1186/s12968-017-0386-y 28954631 PMC5618724

[9] Liu A Wijesurendra RS Francis JM Robson MD Neubauer S Piechnik SK. Adenosine stress and rest T1 mapping can differentiate between ischemic, infarcted, remote, and normal myocardium without the need for gadolinium contrast agents. JACC Cardiovasc Imaging. (2016) 9:27–36. 10.1016/j.jcmg.2015.08.018 26684978 PMC4708879

[10] Burrage MK Shanmuganathan M Masi A Hann E Zhang Q Popescu IA. Cardiovascular magnetic resonance stress and rest T1-mapping using regadenoson for detection of ischemic heart disease compared to healthy controls. Int J Cardiol. (2021) 333:239–45. 10.1016/j.ijcard.2021.03.010 33705843 PMC8117972

[11] Burrage MK Shanmuganathan M Zhang Q Hann E Popescu IA Soundarajan R. Cardiac stress T1-mapping response and extracellular volume stability of MOLLI-based T1-mapping methods. Sci Rep. (2021) 11:13568. 10.1038/s41598-021-92923-4 34193894 PMC8245629

[12] Look DC Locker DR. Time saving in measurement of NMR and EPR relaxation times. Rev Sci Instruments. (1970) 41:250–1. 10.1063/1.168448224313031

[13] Piechnik SK Ferreira VM Dall'Armellina E Cochlin LE Greiser A Neubauer S. Shortened Modified Look-Locker Inversion recovery (ShMOLLI) for clinical myocardial T1-mapping at 1.5 and 3 T within a 9 heartbeat breathhold. J Cardiovasc Magn Reson. (2010) 12:69. 10.1186/1532-429X-12-69 21092095 PMC3001433

[14] Messroghli DR Plein S Higgins DM Walters K Jones TR Ridgway JP. Human myocardium: single-breath-hold MR T1 mapping with high spatial resolution—reproducibility study. Radiology. (2006) 238:1004–12. 10.1148/radiol.2382041903 16424239

[15] Kellman P Wilson JR Xue H Ugander M Arai AE. Extracellular volume fraction mapping in the myocardium, part 1: evaluation of an automated method. J Cardiovasc Magn Reson. (2012) 14:63. 10.1186/1532-429X-14-63 22963517 PMC3441905

[16] Chefd'hotel C Hermosillo G Faugeras O. Flows of diffeomorphisms for multimodal image registration. In: Proceedings IEEE International Symposium on Biomedical Imaging. (2002). p. 753–6. 10.1109/ISBI.2002.102936727295638

[17] Xue H Shah S Greiser A Guetter C Littmann A Jolly MP. Motion correction for myocardial T1 mapping using image registration with synthetic image estimation. Magn Reson Med. (2012) 67:1644–55. 10.1002/mrm.23153 22135227 PMC4158317

[18] Zhou R Huang W Yang Y Chen X Weller DS Kramer CM. Simple motion correction strategy reduces respiratory-induced motion artifacts for k-t accelerated and compressed-sensing cardiovascular magnetic resonance perfusion imaging. J Cardiovasc Magn Reson. (2018) 20:6. 10.1186/s12968-018-0427-1 29386056 PMC5793398

[19] Becker KM Blaszczyk E Funk S Nuesslein A Schulz-Menger J Schaeffter T. Fast myocardial T1 mapping using cardiac motion correction. Magn Reson Med. (2020) 83:438–451. 10.1002/mrm.27935 31418924

[20] Robinson AA Chow K Salerno M. Myocardial T1 and ECV Measurement. JACC Cardiovasc Imaging. (2019) 12:2332–44. 10.1016/j.jcmg.2019.06.031 31542529 PMC7008718

[21] Schulz-Menger J Bluemke DA Bremerich J Flamm SD Fogel MA Friedrich MG. Standardized image interpretation and post-processing in cardiovascular magnetic resonance - 2020 update. J Cardiovasc Magn Reson. (2020) 22:19. 10.1186/s12968-020-00610-6 32160925 PMC7066763

[22] Leiner T Rueckert D Suinesiaputra A Baeßler B Nezafat R Išgum I. Machine learning in cardiovascular magnetic resonance: basic concepts and applications. J Cardiovasc Magn Reson. (2019) 21:61. 10.1186/s12968-019-0575-y 31590664 PMC6778980

[23] Fu Y Lei Y Wang T Curran WJ Liu T Yang X. Deep learning in medical image registration: a review. Phys Med Biol. (2020) 65:20TR01. 10.1088/1361-6560/ab843e 32217829 PMC7759388

[24] Petersen SE Matthews PM Bamberg F Bluemke DA Francis JM Friedrich MG. Imaging in population science: cardiovascular magnetic resonance in 100,000 participants of UK Biobank - rationale, challenges and approaches. J Cardiovasc Magn Reson. (2013) 15:46. 10.1186/1532-429X-15-46 23714095 PMC3668194

[25] Piechnik SK Jerosch-Herold M. Myocardial T1 mapping and extracellular volume quantification: an overview of technical and biological confounders. Int J Cardiovasc Imaging. (2018) 34:3–14. 10.1007/s10554-017-1235-7 28849419 PMC5851695

[26] Ronneberger O Fischer P Brox T. U-Net: convolutional networks for biomedical image segmentation. In: Navab N Hornegger J Wells WM Frangi AF editors. Medical Image Computing and Computer-Assisted Intervention-MICCAI. (2015). Cham: Springer International Publishing (2015). p. 234–41. 10.1007/978-3-319-24574-4_28

[27] Sun D Yang X Liu M Kautz J. PWC-Net: CNNs for optical flow using pyramid, warping, and cost volume. In 2018 IEEE/CVF Conference on Computer Vision and Pattern Recognition (CVPR). Los Alamitos, CA: IEEE Computer Society (2018). p. 8934–43. 10.1109/CVPR.2018.00931

[28] Sokooti H De Vos B Berendsen F Lelieveldt BP Išgum I Staring M. Nonrigid image registration using multi-scale 3D convolutional neural networks. In: Descoteaux M Maier-Hein L Franz A Jannin P Collins DL Duchesne S editors. Medical Image Computing and Computer-Assisted Intervention-MICCAI 2017. Cham: Springer International Publishing (2017). p. 232–9. 10.1007/978-3-319-66182-7_27

[29] Werys K Dragonu I Zhang Q Popescu I Hann E Puchta H. Total mapping toolbox (TOMATO): an open source library for cardiac magnetic resonance parametric mapping. SoftwareX. (2020) 11:100369. 10.1016/j.softx.2019.100369

[30] Onofrey JA Casetti-Dinescu DI Lauritzen AD Sarkar S Venkataraman R Fan RE. Generalizable multi-site training and testing of deep neural networks using image normalization. in 2019 IEEE 16th International Symposium on Biomedical Imaging (ISBI 2019). (2019). p. 348–51. 10.1109/ISBI.2019.8759295 32874427 PMC7457546

[31] Kingma DP Ba J. Adam: a method for stochastic optimization. arXiv e-prints. (2014).

[32] Abadi M Agarwal A Barham P Brevdo E Chen Z Citro C. TensorFlow: large-scale machine learning on heterogeneous distributed systems. arXiv e-prints. (2016).

[33] Papież BW Heinrich MP Fehrenbach J Risser L Schnabel JA. An implicit sliding-motion preserving regularisation via bilateral filtering for deformable image registration. Med Image Anal. (2014) 18:1299–311. 10.1016/j.media.2014.05.005 24968741

[34] Cochran WG. The combination of estimates from different experiments. Biometrics. (1954) 10:101–29. 10.2307/30016663001666

[35] Lee CH Cook S Lee JS Han B. Comparison of two meta-analysis methods: inverse-variance-weighted average and weighted sum of z-scores. Genomics Inform. (2016) 14:173–180. 10.5808/GI.2016.14.4.173 28154508 PMC5287121

[36] Armstrong RA. When to use the Bonferroni correction. Ophthalmic Physiol Optics. (2014) 34:502–8. 10.1111/opo.12131 24697967

[37] Willemink MJ Koszek WA Hardell C Wu J Fleischmann D Harvey H. Preparing medical imaging data for machine learning. Radiology. (2020) 295:4–15. 10.1148/radiol.2020192224 32068507 PMC7104701

[38] Piechnik SK Neubauer S Ferreira VM. State-of-the-art review: stress T1 mapping–technical considerations, pitfalls and emerging clinical applications. Magn Reson Mater Phys Biol Med. (2018) 31:131–41. 10.1007/s10334-017-0649-5 28914389 PMC5813075

[39] Bergamino M Bonzano L Levrero F Mancardi GL Roccatagliata L. A review of technical aspects of T1-weighted dynamic contrast-enhanced magnetic resonance imaging (DCE-MRI) in human brain tumors. Phys Med. (2014) 30:635–43. 10.1016/j.ejmp.2014.04.005 24793824

[40] Jaubert O Arrieta C Cruz G Bustin A Schneider T Georgiopoulos G. Multi-parametric liver tissue characterization using MR fingerprinting: Simultaneous T1, T2, T2^*^, and fat fraction mapping. Magn Reson Med. (2020) 84:2625–35. 10.1002/mrm.28311 32406125

[41] Zheng JQ Lim NH Papież BW. D-net: siamese based network for arbitrarily oriented volume alignment. In: Reuter M Wachinger C Lombaert H Paniagua B Goksel O Rekik I editors. Shape in Medical Imaging. Cham: Springer International Publishing (2020). p. 73–84. 10.1007/978-3-030-61056-2_6

[42] Zhang Q Hann E Werys K Wu C Popescu IA Lukaschuk E. Deep learning with attention supervision for automated motion artefact detection in quality control of cardiac T1-mapping. Artif Intell Med. (2020) 110:101955. 10.1016/j.artmed.2020.101955 33250143 PMC7718111

[43] Lara Hernandez KA Rienmüller T Baumgartner D Baumgartner C. Deep learning in spatiotemporal cardiac imaging: a review of methodologies and clinical usability. Comput Biol Med. (2021) 130:104200. 10.1016/j.compbiomed.2020.104200 33421825

[44] Hann E Piechnik S Popescu IA Zhang Q Werys K Ferreira V. Method and Apparatus for Quality Prediction. WIPO: Patent WO 2020/161481 A1 (2020).

[45] Hann E Popescu IA Zhang Q Gonzales RA Barutçu A Neubauer S. Deep neural network ensemble for on-the-fly quality control-driven segmentation of cardiac MRI T1 mapping. Med Image Anal. (2021) 71:102029. 10.1016/j.media.2021.102029 33831594 PMC8204226

[46] Hann E Gonzales RA Popescu IA Zhang Q Ferreira VM Piechnik SK. Ensemble of deep convolutional neural networks with monte carlo dropout sampling for automated image segmentation quality control and robust deep learning using small datasets. In: Papie z BW Yaqub M Jiao J Namburete AIL Noble JA editors. Medical Image Understanding and Analysis. Cham: Springer International Publishing (2021). p. 280–93. 10.1007/978-3-030-80432-9_22

[47] Zhang Q Piechnik S Werys K Popescu IA Ferreira V. Validation of Quantitative Magnetic Resonance Imaging Protocols. WIPO: Patent WO 2020/234570 A1 (2020).

[48] Zhang Q Werys K Popescu IA Biasiolli L Ntusi NAB Desai M. Quality assurance of quantitative cardiac T1-mapping in multicenter clinical trials — A T1 phantom program from the hypertrophic cardiomyopathy registry (HCMR) study. Int J Cardiol. (2021) 330:251–8. 10.1016/j.ijcard.2021.01.026 33535074 PMC7994017

